# Anti-inflammatory and cognitive effects of interferon-β1a (IFNβ1a) in a rat model of Alzheimer’s disease

**DOI:** 10.1186/s12974-019-1417-4

**Published:** 2019-02-18

**Authors:** Giuseppa Mudò, Monica Frinchi, Domenico Nuzzo, Pietro Scaduto, Fulvio Plescia, Maria F. Massenti, Marta Di Carlo, Carla Cannizzaro, Giovanni Cassata, Luca Cicero, Maria Ruscica, Natale Belluardo, Luigi M. Grimaldi

**Affiliations:** 1 Department of Biomedicine, Neuroscience and Advanced Diagnostics, Division of Human Physiology, University of Palermo, 90134 Palermo, Italy; 2 Institute of Biomedicine and Molecular Immunology “Alberto Monroy” (IBIM), Consiglio Nazionale delle Ricerche (CNR), 90146 Palermo, Italy; 3 Department of Sciences for Health Promotion and Mother and Child Care “Giuseppe D’Alessandro”, University of Palermo, 90134 Palermo, Italy; 4 Experimental Zooprophylactic Institute of Sicily “A. Mirri”, Palermo, Italy; 5 Neurology Department, Fondazione Istituto Giuseppe Giglio, Cefalù, PA Italy

**Keywords:** IFNβ1a, Neuroinflammation, ROS, Pro-inflammatory cytokines, SOD, Aβ_1-42_, IL-10, NF-kB, Hippocampus

## Abstract

**Background:**

Aβ_1-42_ peptide abnormal production is associated with the development and maintenance of neuroinflammation and oxidative stress in brains from Alzheimer disease (AD) patients. Suppression of neuroinflammation may then represent a suitable therapeutic target in AD. We evaluated the efficacy of IFNβ1a in attenuating cognitive impairment and inflammation in an animal model of AD.

**Methods:**

A rat model of AD was obtained by intra-hippocampal injection of Aβ_1-42_ peptide (23 μg/2 μl). After 6 days, 3.6 μg of IFNβ1a was given subcutaneously (s.c.) for 12 days. Using the novel object recognition (NOR) test, we evaluated changes in cognitive function. Measurement of pro-inflammatory or anti-inflammatory cytokines, reactive oxygen species (ROS), and SOD activity levels was performed in the hippocampus. Data were evaluated by one-way ANOVA with Fisher’s Protected Least Significant Difference (PLSD) test.

**Results:**

We showed that treatment with IFNβ1a was able to reverse memory impairment and to counteract microglia activation and upregulation of pro-inflammatory cytokines (IL-6, IL-1β) in the hippocampus of Aβ_1-42_-injected rats. The anti-inflammatory cytokine IL-10, significantly reduced in the Aβ_1-42_ animals, recovered to control levels following IFNβ1a treatment. IFNβ1a also reduced ROS and lipids peroxidation and increased SOD1 protein levels in the hippocampus of Aβ_1-42_-injected rats.

**Conclusion:**

This study shows that IFNβ1a is able to reverse the inflammatory and cognitive effects of intra-hippocampal Aβ_1-42_ in the rat. Given the role played by inflammation in AD pathogenesis and the established efficacy of IFNβ1a in the treatment of inflammatory diseases of the central nervous system such as multiple sclerosis, its use may be a viable strategy to inhibit the pro-inflammatory cytokine and oxidative stress cascade associated with Aβ deposition in the hippocampus of AD patients.

## Background

Alzheimer disease (AD), an age-dependent neurodegenerative disorder and the most common cause of dementia, is a multifactorial disease with a complex interplay of genetics and environmental factors [1, 2]. The pathological hallmarks of AD include the deposition of extracellular neuritic plaques of β-amyloid (Aβ) peptide and the formation of intracellular neurofibrillary tangles in the brain [3]. Aβ peptides are neurotoxic and may cause neurodegenerative changes, including apoptosis, oxidative stress, and neuroinflammation. Neuroinflammation has been widely recognized as a possible pathological contributor to AD, usually including activation of glial cells, such as microglia and astrocytes [4], and release of cytotoxic compounds, e.g., cytokines and ROS, able to cause neuronal damage and death [3, 5]. Since suppression of neuroinflammation could represent an interesting therapeutic target for AD, several approaches have been tested to smolder inflammatory processes by using anti-inflammatory drugs [5–8] or other drugs with anti-inflammatory effects in AD models [9]. However, although all anti-inflammatory strategies tried so far in AD patients have not achieved satisfactory results, indicating the need for a better understanding of the role of the immune system in cerebral proteinopathies and how to modulate it [10], intervention with drugs modulating pro-inflammatory cytokine production is still considered a potentially useful strategy to slow down the disease course of this dreadful disease.

Interferons (IFNs) are a super-family of cytokine proteins that play an important role in the host immune response to infections and immune-mediated diseases [11]. Interferon-β1a (IFNβ1a) is the prototypical regulatory cytokine with anti-inflammatory properties largely used in the past two decades to slow down pathological and clinical features of central nervous system (CNS) immune-mediated diseases, such as multiple sclerosis [12–17] and its animal model experimental autoimmune encephalomyelitis [18, 19]. IFNβ-1a displays several cellular and humoral immune effects, including inhibition of pro-inflammatory cytokine (IL-6, IL-1β, TNF-α, IFN-γ) and downregulation of glial cells and oxidative stress [20]. Interestingly, IFNβ1a significantly prevented cognitive decline in a large cohort of patients with multiple sclerosis, thus suggesting that modulation of neuroinflammatory pathways may prevent cognitive decline in humans [21, 22], as well as cortical atrophy associated with cognitive impairment in patients with multiple sclerosis [23]. We also preliminarily evaluated the safety and efficacy of IFNβ1a in subjects affected by mild-to-moderate AD in a phase 2a study mainly aimed to evaluate the safety of the drug in this elderly population [24], and although not statistically significant, we observed a reduction in disease progression during follow-up as measured by the AD Physical Self-Maintenance Scale.

Taken together, these human studies support the hypothesis that IFNβ1a could attenuate the inflammatory response in AD and led us to look for an experimental confirmation by assessing the efficacy of IFNβ1a in attenuating inflammation in an animal model of AD obtained by intra-hippocampal injection of Aβ_1-42_ peptide. Intra-hippocampal injection of Aβ_1-42_ peptide is considered a suitable animal model of AD [25] with several pathological and behavioral features of AD patients, including cognitive impairment and inflammatory reactivity [26]. Therefore, we first evaluated in rats the cognitive impairment induced by intra-hippocampal injection of Aβ_1-42_ peptide and the therapeutic effects of IFNβ1a treatment. The anti-inflammatory effects of IFNβ1a were then assessed by examining in post-mortem rat brains several inflammation markers, such as pro- and anti-inflammatory cytokines, and oxidative stress responses.

## Methods

### Animals

Adult female Wistar rats (3 months old) were used. Rats were housed in a specific pathogen-free environment, three per polypropylene cages in controlled temperature (23 ± 2 °C), humidity (50–55%), and light (12-h light/dark cycle), with access to food and water ad libitum. Procedures involving animal were carried out in accordance with the Italian institutional guidelines (D. LGS. no. 26, GU n.61, March 2014). All applicable international, national, and/or institutional guidelines for the care and use of animals were followed. No other methods to perform the described experiments (3Rs) were found.

### Experimental design

For this study, we used the following experimental groups: (1) a control group treated with saline only (control); (2) a sham-operated control group and treated with saline (sham); (3) an Aβ_1-42_ peptide-treated group; (4) an interferon-β1a (IFNβ1a)-treated group; and (5) an Aβ_1-42_ peptide + IFNβ1a-treated group. Rats received two bilateral intra-hippocampal injections of 23 μg/2 μl of Aβ_1-42_ peptide dissolved in 0.9% physiological saline solution. IFNβ1a was given subcutaneously (s.c.) at a dose of 3.6 μg (1 M Units, Rebif, Merk Serono, London) in a volume of 0.1 ml of 0.9% of saline solution. We used female rats since in AD disproportionately the female/male ratio is 2:1, although the biological basis of these sex-based differences in AD onset and progression remains elusive [27, 28].

### Aβ_1-42_ peptide preparation and toxicity

Aβ_1-42_ protein was produced according to Carrotta et al. [29]. For the kinetics of aggregation, the sample of Aβ_1-42_ protein was loaded in a 96 black multi-well and added with 8 μM of thioflavin-T. The multi-well was read to the plate reader every 30 s at 450–485 nm wavelength for 8 h at 37 °C. After the 8 h of incubation, the formation of Aβ_1-42_ protein aggregates was also evaluated at the fluorescence microscope (Leica Microsystems, Heidelberg, Germany). In addition, the mean size of the Aβ_1-42_ plates was measured by fluorescence microscopy software (Leica QFluoro V1.1 software, Heidelberg, Germany).

LAN5 cells were cultured with RPMI 1640 medium (Celbio srl, Milan, Italy) supplemented with 10% fetal bovine serum (Gibco-Invitrogen, Milan, Italy) and 1% antibiotics (50 mg mL − 1 penicillin and 50 mg ml − 1 streptomycin). Cells were maintained in a humidified 5% CO_2_ atmosphere at 37 ± 0.1 °C. For dose-effect studies of Aβ_1-42_ toxicity, cells were treated with 50, 75, and 100 μM of Aβ_1-42_ for 24 h, and thereafter, their viability was evaluated by MTS assay ([3-(4,5-dimethylthiazol-2-yl)-5-(3-carboxymethoxyphenyl)-2-(4-sulphophenyl)-2H-tetrazolium]; Promega Italia, S.r.l., Milan, Italy) and morphological analyses. After 24 h of cell treatment with Aβ_1-42_, 20 μl of the MTS solution was added to each well for 3 h at 37 °C, 5% CO_2_. The absorbance was read at 490 nm on the Microplate reader (WallacVictor 2 1420 Multilabel Counter) (PerkinElmer, Inc. Monza, Italy).

### Aβ_1-42_ intra-hippocampal injection

The Aβ_1-42_ peptide intra-hippocampal injection was performed as described by Mudò et al. [30]. Shortly, rats were anesthetized with mixture of 1:1 of Zolazepam + tiletamine 15 mg/kg b.w. (Zoletil, Virbac) and xilazine 9 mg/kg b.w. (Nerfasin, ATI, Italy), placed in a David Kopf stereotaxic apparatus, and received two bilateral intra-hippocampal injections of 23 μg/2 μl of Aβ_1-42_ peptide, using the following stereotaxic coordinates from the Bregma, according to Paxinos and Watson (1998): first injection AP = − 3.6, *L* = 2, and *V* = 4.5; second injection AP = − 4.2, *L* = 2.4, and *V* = 4.5. The sham group was intra-hippocampal-injected with 2 μl of 0.9% physiological saline. Injections were performed by 30-gauge injector cannula that was connected by a piece of polyethylene tube to the 10 μl Hamilton syringe. Each injection was performed over 3 min, and following injection, the needle remained in the target location for 3 min to avoid Aβ_1-42_ peptide reflux along the needle tract and to achieve a proper diffusion of the drug. After surgery, each rat was treated with penicillin (100,000 U/i.m.) to prevent infection.

### Behavioral testing

Using the novel object recognition (NOR) test, we evaluated changes in cognitive function of the experimental animals. Rats were tested in an open field Plexiglas square box, in a mean light intensity (100 lx) illuminated chamber. All experimental groups were subjected to a 5-min training session when they were presented two identical non-toxic objects (i.e., two metal cans) placed against a wall in the open field arena. To prevent coercion to explore the objects, rats were released against the opposite wall with the back to the objects. The time spent on exploring each object was recorded using ANY MAZE Video Tracking System (Ugo Basile, Italy); a 2-cm^2^ area surrounding the objects was defined such that nose entries were recorded as time exploring the objects. After the training session, animals were placed in their home cage for a 24-h retention interval. Then, they were returned to the arena containing two objects: one was identical to the familiar one but previously unused (to prevent olfactory cues and the necessity to wash objects during experimentation) and the other was a novel object (metal, glass, or hard plastic items). Time spent on exploring each object was recorded along 5-min session. Objects were randomized and counterbalanced across animals. The objects and arena were thoroughly cleaned at the end of each experimental session. The recognition index (RI), which is the time spent on investigating the novel object, divided by the total amount of exploration time of the novel (TN) and familiar objects (TF), [RI = TN/(TN + TF)], is a measure of novel object recognition and the main index of retention. If RI percentage is higher than 50%, it indicates more time spent on inquiring into the novel object, whereas less than 50% indicates that time was prevailingly spent on exploring the familiar object, and 50% indicates a null preference.

### Immunohistochemistry

The rats under deep anesthesia were perfused through the aorta with 0.9% saline and brain dissected. The left side of the brain was used for histological investigations and the right side for molecular analysis. Immuno-histochemical investigation was performed as described by Di Liberto et al. [31]. The left side of brain was fixed with 4% of paraformaldehyde in 0.1 M phosphate buffer (pH 7.4) for 2 days and then immersed in the sucrose 10% solution for 1 day and in sucrose 20% for 2 days. Subsequently, brains were frozen in cooled isopentane and 20-μm-thick coronal sections at hippocampal level were prepared and processed for immunohistochemistry as free-floating sections. Sections were washed for 5 min in 0.1 M PBS and incubated for 15 min with BSA (5 mg/ml) and Triton X-100 (0.3%) in PBS 0.1 M. Mouse monoclonal antibody anti-glial fibrillary acidic protein (anti-GFAP; diluted 1:400; MAB360 Chemicon) or rabbit anti-ionized calcium-binding adapter molecules-1 (anti-Iba-1; diluted 1:300 Wako Catalog No. 019-19741) was added to sections that were then incubated at 4 °C overnight. After two washing steps with PBS for 5 min, the sections were incubated at RT for 1 h with specific Cy2-conjugated secondary antibodies diluted 1:250 (711225152 and 115-165-003; Jackson Immuno Research, West Grove, PA, USA). Following two washing steps with PBS, the sections were counterstained by incubation for 10 min in 0.5 mg/ml of the fluorescent nuclear dye Hoechst 33258 (bisbenzimide, Sigma–Aldrich, Seelze, Germany). Following a short washing with PBS, sections were coverslipped in a glycerol-based medium and slides were examined under a fluorescence microscope (DMRBE, Leica Microsystems, Wetzlar, Germany).

### Western blotting

Rats were sacrificed at the end of experimental procedures by an overdose of anesthesia, and the hippocampi were rapidly removed from their brain, collected, and stored at − 70 °C for later use. Dissected hippocampal tissue was homogenized in cold radio-immunoprecipitation assay (RIPA) buffer (50 mM Tris, pH 7.4, 150 mM NaCl, 1% Triton, SDS 0.1%), supplemented with protease inhibitor cocktail (Sigma-Aldrich P8340) and phosphatase inhibitor cocktail (Sigma–Aldrich P5726). Samples were sonicated (30 pulsations/min), quantified by the Lowry method [32], and stored at − 80 °C. Western blotting was performed as previously described by Frinchi et al. [33]. Protein samples (50 μg per lane) and molecular weight marker (161-0376 BIO-RAD) were run on 10% or 12% polyacrylamide gel and electrophoretically transferred onto nitrocellulose membrane (RPN303E, Hybond-C-extra, GE Healthcare Europe GmbH). The membranes were incubated for 1 h in blocking buffer, 1x TBS, 0.1% Tween-20, and 5% *w*/*v* nonfat dry milk, with gentle shaking overnight at − 4 °C with specific antibody in blocking buffer. For detection of superoxide dismutase-1 (SOD1) and superoxide dismutase-2 (SOD2), the following antibodies were used: rabbit polyclonal anti-SOD1 1:1000 (Sc-11407 Santa Cruz Biotechnology); mouse anti-SOD2 1:500 (SOD2; sc-137254, Santa Cruz Biotechnology). For detection of GFAP and Iba-1, the following antibodies were used: mouse monoclonal antibody anti-GFAP 1:2000 (MAB360 Chemicon), rabbit anti-Iba-1 1:1000 (Wako Catalog No. 019-19741). The day after, the membranes were washed three times for 10 min with TBS/T and then incubated for 1 h at room temperature with goat anti-rabbit IgG (sc-2004 Santa Cruz Biotechnology) or goat anti-mouse IgG (Sc.7076 Cell Signaling Technology) horseradish peroxidase-conjugated diluted 1:10000. After three washings with TBS-T, immune complexes were visualized with a chemiluminescence reagent (RPN2236, GE Healthcare Europe GmbH) according to the manufacturer’s instructions. The Hyperfilm (ECL-films 28906837, GE Healthcare Europe GmbH) were developed using Kodak developer and fixer (catalog no. 1900984 and 1902485, Kodak GBX, Eastman Kodak). For the normalization of quantitative evaluation of bands, each membrane was stripped at 65 °C for 30 min in buffer containing NaCl 137 mM, TrisHCl 20 mM pH 7.6, and β-mercaptoethanol 0.01%. After two washings with TBST, the membranes were reprobed with an anti-β-actin antibody (sc-47778, Santa Cruz Biotechnology). The densitometric evaluation of bands was performed by measuring the optical density (O.D.) using the Image J software (Rasband WS, ImageJ, U.S. National Institutes of Health, Bethesda, Maryland, USA, https://imagej.nih.gov/ij/, 1997–2018).

### Measurement of pro-inflammatory or anti-inflammatory cytokines by ELISA assay

Concentrations of interleukin-1β (IL-1β), interleukin-6 (IL-6), interleukin-10 (IL-10), and transforming growth factor-beta1 (TGF-β1) were measured in the hippocampus homogenates (20 mg of tissue sample) using enzyme-linked immunosorbent assay (ELISA) kits for rat (Cloud-Clone Corp, Wuhan, Hubei) according to the manufacturers’ protocols and as reported by Zizzo et al. [34].

### Reactive oxygen species analysis

To assess reactive oxygen species (ROS) generation by fluorimeter analysis, 10 mg of tissue from rat hippocampus was weighed and suspended on 1000 μl of PBS1X with 10 μ of protease inhibitors (Amersham Life Science, Munich, Germany). Samples were then incubated with 1 mM dichlorofluorescein diacetate (DCFH-DA) for 10 min at room temperature in the dark. The conversion of non-fluorescent DCFH-DA to the highly fluorescent compound 20,70-dichlorofluorescein (DCF) by esterase activity was used to monitor the presence of peroxides due to the oxidative burst in the brain [34]. The samples were analyzed by fluorimeter (Microplate reader WallacVictor 2 - 1420 Multilabel Counter; PerkinElmer, Inc.), using the excitation filter set at 485 nm and the emission filter set at 530 nm. Relative ROS production was expressed as the change in fluorescence of the experimental groups compared with that of the control group (100%).

### SOD activity levels

The hippocampus of rats was homogenized in PBS with protease inhibitors (Amersham Life Science, Munich, Germany). To remove insoluble material, tissue lysates were sonicated and centrifuged (14,000 rpm, at 4 °C, for 30 min). In the supernatant, total proteins were quantified by the Lowry method [32]. Volume corresponding to 50 μg of protein was used for total SOD enzymatic activity measurement, by using the SOD assay kit (Sigma–Aldrich) according to manufacturer’s instructions [34]. Absorbance was measured by using the iMark™ Microplate Absorbance Reader at 450 nm.

### Lipid peroxidation assay

The lipid peroxidation assay kit (Sigma Aldrich) was used to detect the concentration of malondialdehyde (MDA), a final product of lipids peroxidation. Ten milligrams of hippocampal tissues was homogenized in 300 μl of MDA lysis buffer (supplied in the kit), and colorimetric reaction with thiobarbituric acid (TBA) was read on an iMark™ Microplate Absorbance Reader at 532 nm, according to manufacturer’s instructions [34].

### Cell counting

The number of Iba-1 and GFAP-positive cells was estimated by counts made by systematic sampling of brain sections, every third section of total 10 sections, in the hippocampal region of rat brain. All counts were made in four rats for each group and were carried out double-blindly. Labeled cells were evaluated using ImageJ software (Cell Counter plugin; Rasband, W.S., ImageJ, U.S. National Institutes of Health, Bethesda, Maryland, USA, http://imagej.nih.gov/ij/, 1997–2018). The cell count values obtained from three to five random fields per section, in 10 sections examined, were expressed as means ± SEM values per square millimeter of tissue.

### Cortisol levels

Rats under anesthesia were sacrificed between 11:00 and 12:00 am, and blood was taken by intracardiac puncture and collected in tubes coated with EDTA. Blood samples were centrifuged at 4000×*g* at 4 °C for 10 min, and the supernatant was stored at − 80 °C. The plasma levels of cortisol were measured using an automated electrochemiluminescence immunoassay (Roche Diagnostics Elecsys Cortisol II assays and COBAS E801), and values were expressed in nanograms per milliliter. The minimum level of detection for assays of cortisol was 0.15 ng/ml [35].

### Statistical analysis

Data analysis was performed using the GraphPad Prism 6 software (GraphPad Software, Inc., La Jolla, CA, USA). Results are presented as mean ± SE, and in some cases are expressed as arbitrary units, with controls equal to 1, or as percentage of control. For the *novel object recognition task*, the parameter chosen to assess rats’ ability to discriminate novelty from familiar features was the recognition index (RI) and was calculated using the following formula: [RI = TN/(TN + TF)]. Statistical evaluations were performed by one-way ANOVA, followed by Fisher’s Protected Least Significant Difference (PLSD) test, with the exclusion of behavioral data for which we used the Tukey’s multiple comparison test. Differences in *P* value less than 0.05 were considered statistically significant.

## Results

### AD rat model

#### Aβ_1-42_ oligomers aggregation and toxicity

**Fig. 1:**
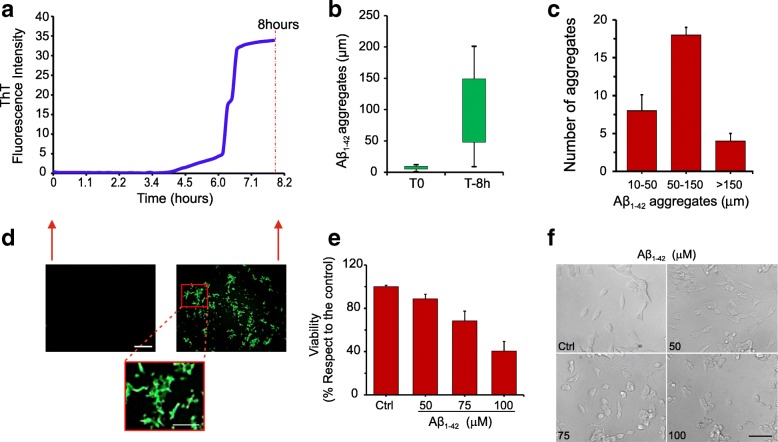
Aβ_1-42_ oligomers aggregation and toxicity. **a**–**c** Aggregation kinetics of Aβ_1-42_ oligomers. **d** Fluorescence imaging of Aβ_1-42_ plates. **e**, **f** Aβ_1-42_ cell toxicity in LAN5 cells treated with 50, 75, and 100 μM of Aβ_1-42_ for 24 h: cell viability (**e**) and dose-effect of cell morphological changes (**f**). Scale: upper panels 100 μm; lower panel 50 μm

Aβ_1-42_ oligomers, prepared as reported in Carrotta et al. [29], were aggregated by incubation for 8 h at 37 °C. The results of aggregation kinetics of Aβ_1-42_ oligomers are shown in Fig. 1a–c. The Aβ_1-42_ oligomers aggregation was also evaluated by fluorescence microscope, and the Aβ_1-42_ plate’s mean size was measured by fluorescence microscopy software; results are shown in Fig. 1d. For Aβ_1-42_ cell toxicity, LAN5 cells were treated with 50, 75, and 100 μM of Aβ_1-42_ for 24 h and the cell viability was evaluated using MTS assay; results are shown in Fig. 1e, f. Based on the present aggregation and toxicity data of Aβ_1-42_ oligomers and on data of neurotoxicity dependent on the types and sizes of Aβ_1-42_ oligomers [36, 37], it was decided to inject Aβ_1-42_ oligomers aggregates, formed after 8 h of incubation and at concentration of 75 μM, in the dorsal hippocampus.

#### Aβ_1-42_ intra-hippocampal injection and IFNβ1a treatment

**Fig. 2:**
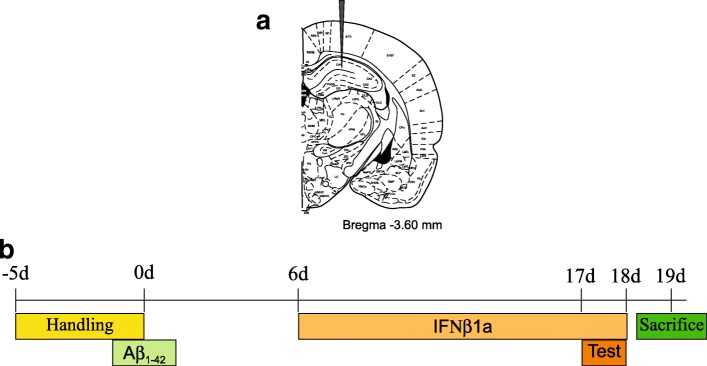
Aβ_1-42_ intra-hippocampal injection and IFNβ1a treatment. **a** Stereotaxic position of Aβ_1-42_ injection in the dorsal hippocampus. **b** Scheme of Aβ_1-42_ and IFNβ1a treatment performed

In Fig. 2a, the stereotaxic position of injection site in the dorsal hippocampus is shown. Fig. 2b shows the scheme of treatment performed. The amount of Aβ_1-42_ protein aggregates injected was 23 μg/2 μl according to toxicity data of previous experimental models [38, 39]. The scheme of dose and time of IFNβ1a treatment was based on previous work using a similar experimental rat model [20] or a rat model of autoimmune encephalomyelitis [40–42].

### IFNβ1a treatment rescues cognitive performances impaired by intra-hippocampal injection of Aβ_1-42_ peptide

**Fig. 3:**
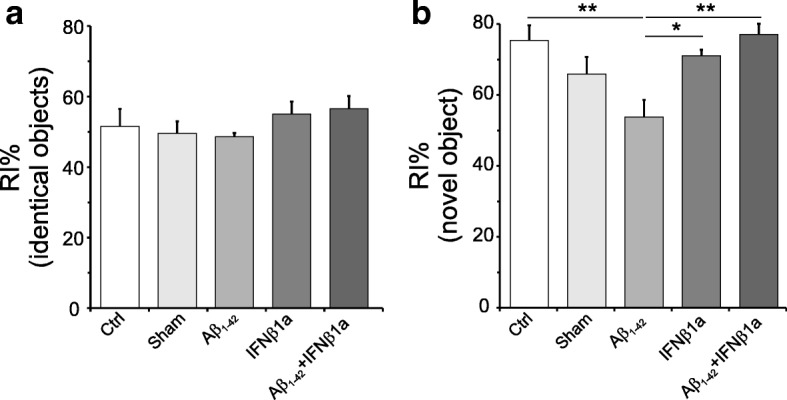
Cognitive evaluation by NOR test. Rats (*n* = 8 per group) exposed to the training with two identical objects did not show any statistical variation in the RI% (*F*_(4,35)_ 0.6122; *p* = 0.6566) among the experimental groups (**a**). Twenty-four hours after the training was the evaluation of rats’ preference toward novel object. The results of one-way ANOVA showed **b** a significant effect of treatment (*F*_(4,35)_ = 5.971; *p* < 0.001). The post hoc analysis conducted by Tukey’s multiple comparison test showed a significant reduction of RI% in the Aβ_1-42_ group with respect to control (*p* < 0.01), Aβ_1-42_ + IFNβ1a (*p* < 0.01), and IFNβ1a (*p* < 0.05) groups (**b**). In the Aβ_1-42_ + IFNβ1a group, the treatment with IFNβ1a full counteracted the RI% reduction observed in the Aβ_1-42_ group. In the IFNβ1a and sham groups, the RI% did not change as compared to control. *P < 0.05, **P < 0.01

Using the NOR test, we evaluated changes in cognitive function induced by intra-hippocampal injection of Aβ_1-42_ peptide. At scheduled time (Fig. 2b), rats were tested in an open field arena in order to assess declarative memory as assessed by the recognition index of novel objects from familiar ones. When rats were trained with two identical objects, a one-way ANOVA did not show (Fig. 3a) any statistical variation in the RI% (*F*_(4,35)_ = 0.6122; *p* = 0.6566) among the experimental groups. Twenty-four hours after the training, rats’ preference toward a novel object was evaluated. A one-way ANOVA showed (Fig. 3b) a significant effect of treatment (*F*_(4,35)_ = 5.971; *p* < 0.001). The post hoc analysis conducted by Tukey’s multiple comparison test showed a significant reduction of RI% in the Aβ_1-42_ group as compared to the control (*p* < 0.01), to Aβ_1-42_ + IFNβ1a (*p* < 0.01), and to IFNβ1a (*p* < 0.05) groups (Fig. 3b). In the Aβ_1-42_ + IFNβ1a group, the treatment with IFNβ1 fully counteracted the RI% reduction observed in the Aβ_1-42_ group. In the IFNβ1a and sham groups, the RI% did not change as compared to control.

### Body weight and cortisol levels

**Fig. 4:**
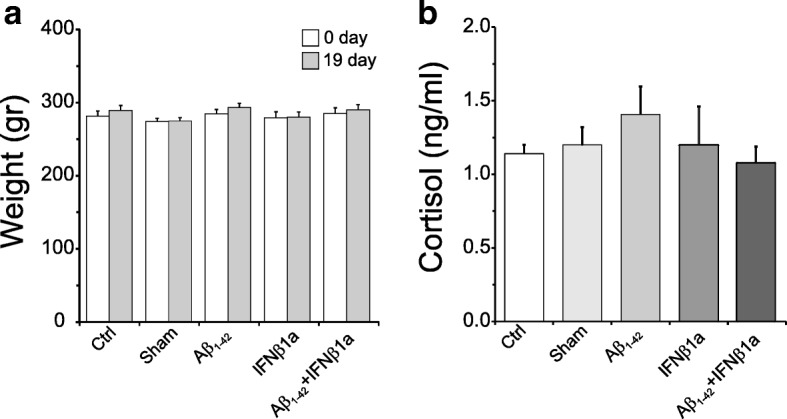
Body weight and cortisol levels. Two-way repeated measurement ANOVA showed not significant differences in the body weight, both among the experimental groups and each group at the end of experimental period as compared to starting body weight (**a**). One-way ANOVA did not show significant change in cortisol level among the experimental groups (**b**)

Rats’ body weight was measured at the beginning and the end of the experimental period (Fig. 2b). Two-way repeated ANOVA measurements showed no significant differences in body weight (Fig. 4a) both among the various experimental groups and in each group at the end of the experimental period as compared to the beginning of the experiment.

Although corticosterone is considered the main glucocorticoid involved in the regulation of stress responses in rodents, we preferred to measure cortisol in consideration of procedure availability in our laboratory and of good correlation between serum cortisol and corticosterone [43]. The results are reported in Fig. 4b: one-way ANOVA did not show significant change in cortisol level among experimental groups.

### IFNβ1a effects on glial cell activation by Aβ_1-42_ protein injection in the hippocampus

**Fig. 5:**
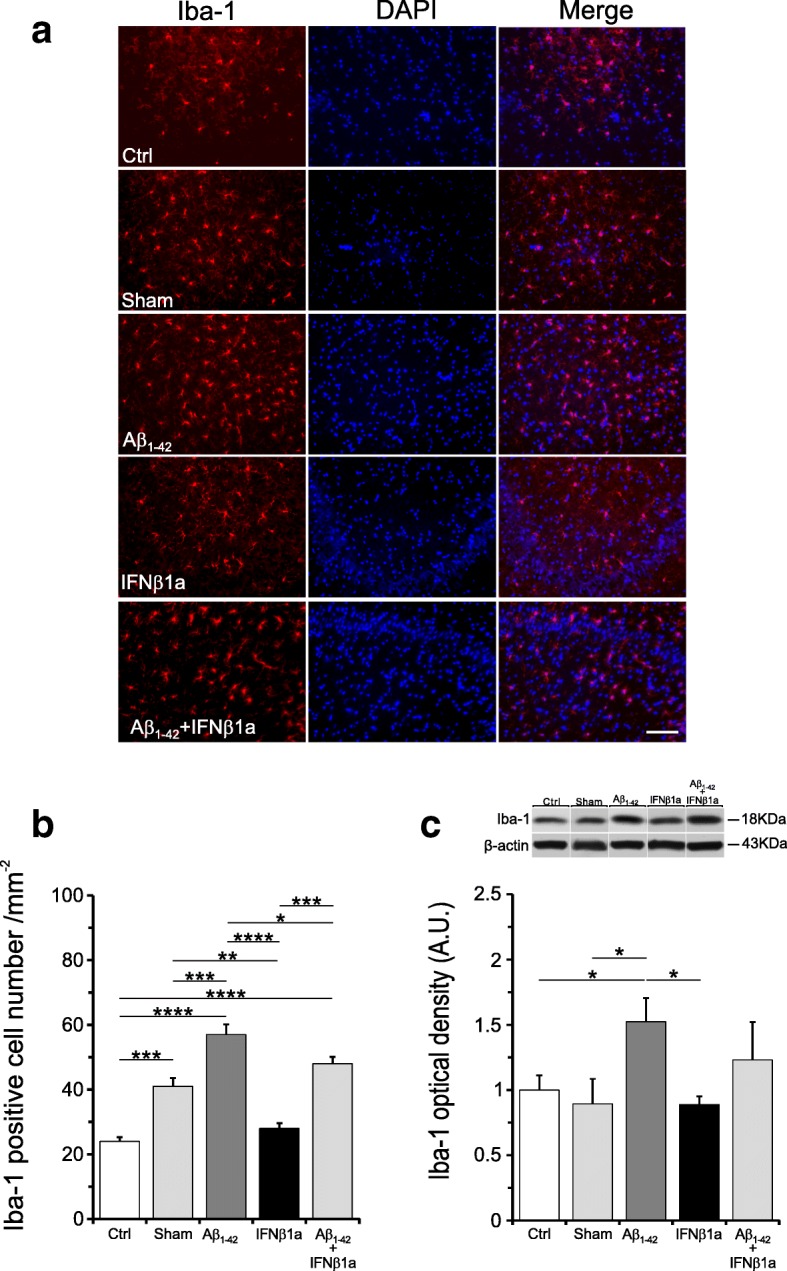
IFNβ1a effects on microglial cell activation by Aβ_1-42_. **a** Iba-1 immunohistochemistry in the hippocampus. **b** Count of Iba-1-positive cells and **c** quantitative Western blot analyses of Iba-1 protein levels. **b** The count of Iba-1-positive cells showed a significant increase of cell number in the hippocampus region of the Aβ_1-42_-treated group (*n* = 6) and treatment with IFNβ1a (*n* = 6) significantly counteract this effect in the Aβ_1-42_+ IFNβ1a group (*n* = 6) as compared to sham group but not to the control group. **c** Similarly, quantitative Western blot analyses of Iba-1 protein levels showed that in the Aβ_1-42_ group, Iba-1 protein levels were significantly increased and treatment with IFNβ1a significantly counteract this effect in the Aβ_1-42_ + IFNβ1a group. Scale: 100 μm. *P < 0.05, **P < 0.01, ***P < 0.001, ****P < 0.0001

Since one of the features of AD pathology is activation of microglia and astrocytes induced by Aβ deposits, we analyzed by immunohistochemistry and Western blot Iba-1 and GFAP markers for microglia and astrocyte activation. As shown in Fig. 5a, b, the count of Iba-1-positive cells was significantly increased in the hippocampus of the Aβ_1-42_-treated group (*F*_(4,22)_ = 26.97, *p* < 0.0001) as compared to the control (*p* < 0.0001) and sham groups (*p* < 0.001). However, in the Aβ_1-42_ + IFNβ1a group, the treatment with IFNβ1a significantly counteracted the effect of Aβ_1-42_ injection as shown by the Iba-1-positive cell number significantly reduced as compared to the Aβ_1-42_ group (*p* = 0.02). However, the cell number in the Aβ_1-42_ + IFNβ1a group was still significantly increased when compared to the control group (*p* < 0.0001) but not compared to the sham group (Fig. 5a, b). The sham group, but not the IFNβ1a group, showed a significant increase of Iba-1-positive cell number as compared to the control group (*p* < 0.001). Quantitative Western blot analyses of Iba-1 protein levels clearly showed a significant increase in the Aβ_1-42_ group as compared to both control (*p* < 0.05) and sham (*p* < 0.05) groups. In the Aβ_1-42_ + IFNβ1a group, the treatment with IFNβ1a counteracted the Aβ_1-42_ effect on Iba-1 protein levels. Indeed, Aβ_1-42_ + IFNβ1a Iba-1 protein levels are not significant when compared to the Aβ_1-42_ group, but they are also not significant when compared both to control and sham groups (Fig. 5c). This means that treatment with IFNβ1a in the Aβ_1-42_ + IFNβ1a group counteracted the increase of Iba-1 protein levels induced by Aβ_1-42_, as shown by its loss of significance with respect to controls, although it does not bring them back to the levels detected in the control and sham groups (Fig. 5c). Iba-1 protein levels in the IFNβ1a and sham groups did not show significant difference as compared to the control group.

**Fig. 6:**
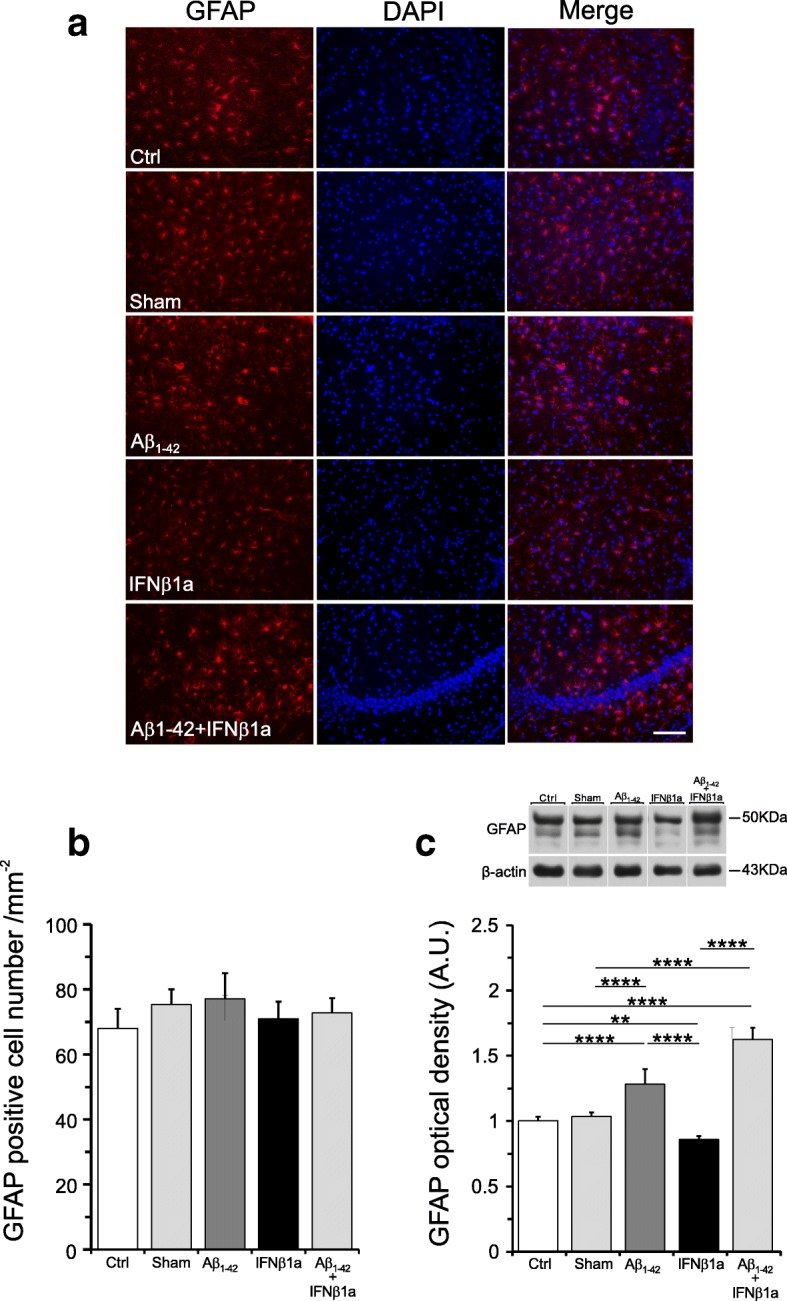
IFNβ1a effects on astroglial cell activation by Aβ1-42. **a** GFAP immunohistochemistry in the hippocampus. **b** Count of GFAP positive cells and **c** quantitative Western blot analyses of GFAP protein levels. **b** Count of astrocytes number showed no significant changes in all experimental groups. **c** By contrast, the quantitative Western blot analyses of GFAP protein levels revealed that in the Aβ_1-42_ group (*n* = 6), GFAP protein levels were significantly increased and this increase was not counteracted by treatment with IFNβ1a (*n* = 6) in the Aβ_1-42_ + IFNβ1a group (*n* = 6). Note IFNβ1a alone significantly reduced the GFAP protein levels as compared to the control group. Scale: 100 μm *P < 0.05, **P < 0.01, ***P < 0.001, *^*^**P < 0.0001

The immunohistochemistry analysis of GFAP marker showed a not significant trend toward an increase in the astrocytes numb in all experimental groups as compared to control group (Fig. 6a, b). By contrast, the quantitative Western blot analyses of GFAP protein levels showed that in the Aβ_1-42_ and in the Aβ_1-42_ + IFNβ1a groups, GFAP protein levels were significantly increased (both *p* < 0.0001) as compared to the control group and in the Aβ_1-42_ + IFNβ1a group as compared to the sham group (Fig. 6c). Surprisingly, the treatment with IFNβ1a alone significantly reduced (*p* < 0.01) the GFAP protein levels as compared to the control group. The cell number of the sham group was not significantly different from the control group.

### IFNβ1a inhibits pro-inflammatory cytokines increase induced by Aβ_1-42_ protein injection in the hippocampus

**Fig. 7:**
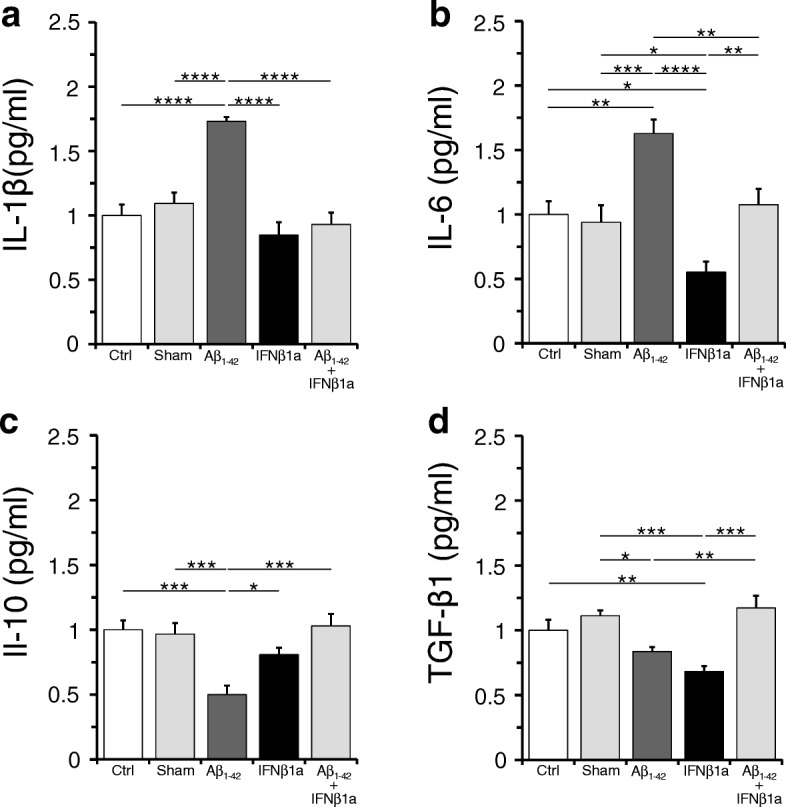
IFNβ1a effects on inflammatory cytokines in the hippocampus. **a**, **b** Hippocampal levels of pro-inflammatory cytokines, IL-1β and IL-6. Note that IL-1β and IL-6 levels were significantly increased in the Aβ_1-42_ group (*n* = 5 respectively) and this increase was counteracted by treatment with IFNβ1 (*n* = 5 respectively) in the Aβ_1-42_ + IFNβ1a group (*n* = 5 respectively). **c**, **d** Hippocampal levels of anti-inflammatory cytokines, IL-10 and TGF-β1. Note IL-10 and TGF-β1 levels showed significant reduction in the Aβ_1-42_ group (*n* = 5 respectively) and following IFNβ1a (*n* = 5, respectively) treatment in the Aβ_1-42_ + IFNβ1a group (*n* = 5, respectively) were recovered to control (*n* = 5 and *n* = 6, respectively) levels. *P < 0.05, **P < 0.01, ***P < 0.001, ****P < 0.0001

We also analyzed by ELISA the hippocampal levels of pro-inflammatory cytokines, IL-1β and IL-6, and anti-inflammatory cytokines, IL-10 and TGF-β1. As shown in Fig. 7a, b, we found that both IL-1β and IL-6 levels were significantly increased in the Aβ_1-42_ group as compared to the control (*p* < 0.0001 and *p* < 0.01 respectively) and sham (*p* < 0.0001 and *p* < 0.001 respectively) groups. In the Aβ_1-42_ + IFNβ1a group, the treatment with IFNβ1a counteracted this Aβ_1-42_ effect on IL-1β and IL-6 levels (*p* < 0.0001 and *p* < 0.01 respectively), bringing them back to the levels of control and sham groups. In the IFNβ1a group, the treatment with IFNβ1a did not change the IL-1β levels as compared to the control, whereas it produced a substantial reduction of IL-6 levels as compared to the control and sham groups (*p* < 0.05).

In contrast to hippocampal upregulation of IL-1 and IL-6 levels, the anti-inflammatory cytokine IL-10 was found significantly reduced in the Aβ_1-42_ group as compared to the control (*p* < 0.001) and sham (*p* < 0.001) groups (Fig. 7c). Following IFNβ1a treatment in the Aβ_1-42_ + IFNβ1a group, the IL-10 levels recovered to control levels, strengthening the anti-inflammatory property of IFNβ1a. The TGF-β1 levels were significantly reduced in the IFNβ1a group as compared to control or sham group (*p* < 0.01 and *p* < 0.001 respectively) and Aβ_1-42_ group as compared to sham (*p* < 0.05) group (Fig. 7d), whereas surprisingly in the Aβ_1-42_ + IFNβ1a group, TGF-β1 levels recovered to the control group levels. In the sham group, we did not find significant changes in both IL-10 and TGF-β1 levels as compared to the control group.

### IFNβ1a treatment effects on ROS levels and SOD1 or SOD2 proteins and activity levels

**Fig. 8:**
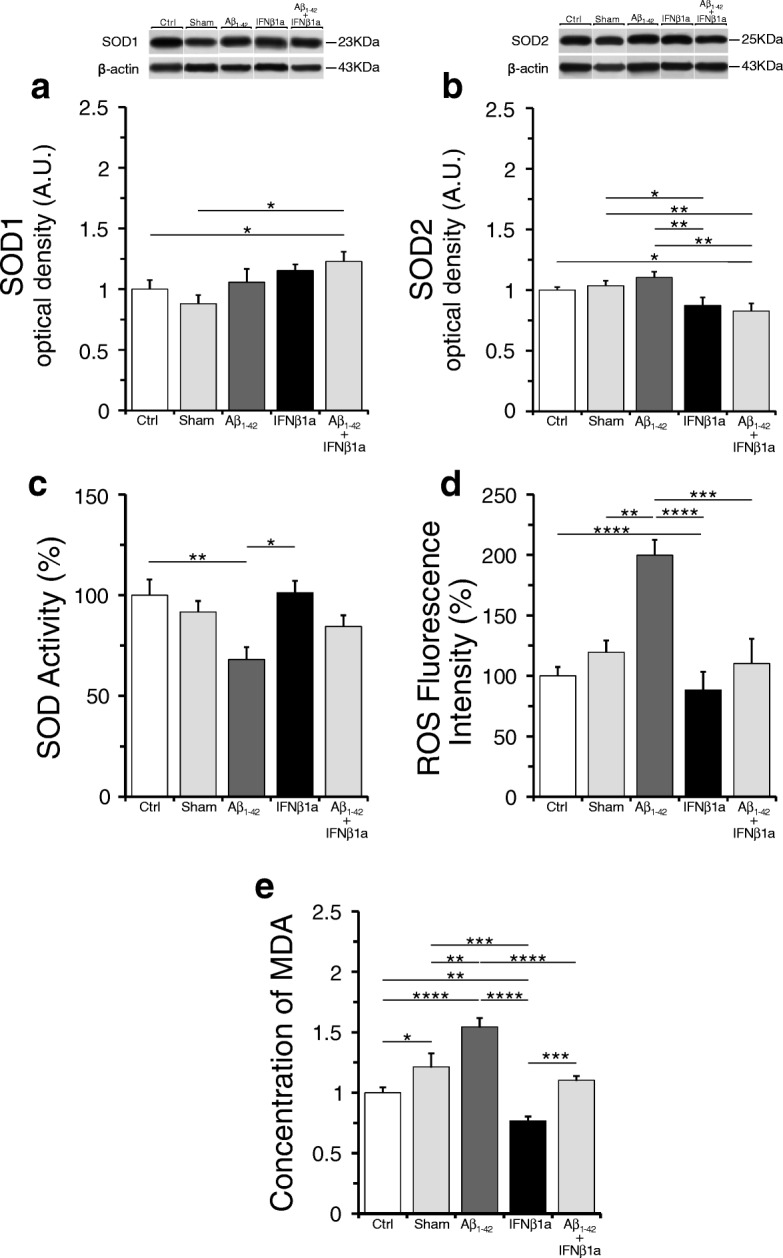
IFNβ1a effects on ROS and lipid peroxidation levels and SOD1 or SOD2 proteins and activity levels in the hippocampus. **a**, **b** The levels of SOD1 showed significant increase in the Aβ_1-42_ + IFNβ1a group (*n* = 5), whereas those of SOD2 appear significantly reduced in the same group (*n* = 5). **c** Total SOD activity levels showed significant decrease in Aβ_1-42_ group (*n* = 5), and this decrease was counteracted in the Aβ_1-42_ + IFNβ1a group (*n* = 5) by treatment with IFNβ1a. **d** ROS levels were significantly increased in the Aβ_1-42_ group (*n* = 5), and this increase was counteracted by treatment with IFNβ1a (*n* = 5). **e** The analysis of lipid peroxidation levels, measured as concentration of MDA, revealed a significant increase of MDA levels in the Aβ_1-42_ group (*n* = 5) that was completely blocked by IFNβ1a treatment in the Aβ_1-42_ + IFNβ1a group (*n* = 5). *P < 0.05, **P < 0.01, ***P < 0.001, ****P < 0.0001

SOD1 levels (Fig. 8a) were increased in the Aβ_1-42_ + IFNβ1a group as compared to the control (*p* < 0.05) and sham (*p* < 0.05) groups, whereas SOD2 levels (Fig. 8b) were significantly reduced in the same group as compared to the control (*p* < 0.05) and sham (*p* < 0.01) groups. The SOD2 levels were significantly lower in the IFNβ1a group as compared to the sham group (*p* < 0.05) but not to the control group, suggesting that IFNβ1a per se may negatively regulate the SOD2 levels. In the sham group, we did not find significant changes in both SOD1 and SOD2 levels as compared to the control group.

Concerning the SOD activity (Fig. 8c), we found that SOD activity levels were significantly decreased in the Aβ_1-42_ group as compared to control (*p* < 0.01). In the Aβ_1-42_ + IFNβ1a group, the treatment with IFNβ1a counteracted the decrease of SOD activity level bringing it back to the control level. In the IFNβ1a group, the treatment with IFNβ1a did not change the SOD activity levels as compared to the control and sham groups. In the sham group, we did not find significant changes in SOD activity levels as compared to the control group.

Concerning the oxidative stress analysis (Fig. 8d), we found that ROS levels were significantly increased in the Aβ_1-42_ group as compared to control (*p* < 0.0001) and sham (*p* < 0.01) groups. In Aβ_1-42_+ IFNβ1a group, the treatment with IFNβ1a counteracted the increase of ROS levels bringing them back to the control level. ROS levels were substantially similar in the IFNβ1a, control, and sham groups.

Lipid peroxidation is the degradation of lipids that occurs as a result of oxidative damage, typically by reactive oxygen species, resulting in a well-defined chain reaction with the production of end products such as MDA. According to previous data [44] showing that Aβ_1-42_ injection induces ROS levels, we analyzed the level of lipid peroxidation in the hippocampus by measuring the concentration of MDA. The analysis (Fig. 8e) revealed a significant increase of MDA levels in the Aβ_1-42_ group as compared to control (*p* < 0.0001) and sham (*p* < 0.01) groups. This increase of MDA levels was positively correlated n with the increased levels of ROS. In the Aβ_1-42_ + IFNβ1a group, the treatment with IFNβ1a completely blocked the increase of MDA levels observed in the Aβ_1-42_ group. Interestingly, in the IFNβ1a group, the MDA levels were found significantly reduced as compared to control (*p* < 0.01) and sham (*p* < 0.001) groups. However, the MDA levels were found also significantly increased in the sham as compared to control group (*p* < 0.05).

## Discussion

In our rat model of AD, the administration of Aβ_1-42_ oligomer aggregates in the dorsal hippocampus led to neuroinflammation, via the activation of glial immune system, and memory impairment as evaluated by the NOR test. The short treatment with IFNβ1a was able to reverse memory impairment and to suppress microglia activation and the upregulation of pro-inflammatory cytokine (IL-6, IL-1β) levels and oxidative stress in the hippocampus. All together, these data suggest a protective effect of IFNβ1a against Aβ_1-42_-induced functional alterations in the hippocampus of our rat AD model.

AD is characterized by an insidious clinical onset, by a progressive cognitive and memory decline, and by accumulation of Aβ plaques and neurofibrillary tangles leading to loss of neurons in the hippocampus and in the cerebral cortex [1–3]. In a previous pilot study, we evaluated the safety and efficacy of IFNβ1a in subjects affected by mild-to-moderate AD and detected a reduction in disease progression and improvements in the instrumental activities of daily living and physical self-maintenance scales [24]. Apart for this limited human study, IFNβ1a had never been previously tested in AD, unlike its long and widespread use in patients with multiple sclerosis, where it has been clearly shown that subcutaneous IFNβ1a protects against cognitive decline [15, 21, 22, 41, 45]. Although most of its pleiotropic effects occur in the peripheral immune system, a direct anti-inflammatory effect of IFNβ1a within the brain has also been proposed [23]. We actually found an anti-inflammatory effect of IFNβ1a in the CNS of experimental rats that reasonably explains the recovery from memory impairment caused by hippocampal Aβ_1-42_ peptide injection. Improvement of memory deficit in AD animal model after treatments targeting inflammation has already been reported [46, 47]. However, it has been reported that microglia of adult mice following chronic exposure to IFNβ1a express an aging-like phenotype and negatively affect learning abilities [48, 49].

Histological studies of brains from individuals with AD have revealed a direct relationship between Aβ_1-42_ peptide abnormal production and the development and/or maintenance of neuro-inflammation and oxidative stress [5, 50]. Accordingly, exposure of the brain to Aβ_1-42_ peptide causes inflammation, by activating microglia as well as astrocytes, and promotes the production of cytotoxic molecules, such as pro-inflammatory cytokines and reactive oxygen species, that contribute to dysfunction, injury, and ultimately neuronal loss [3, 51–55]. In addition, the release of inflammatory mediators may in turn increase Aβ_1-42_ peptide production, which may further contribute to plaque formation and progression to neuronal loss [3, 52]. Besides this consolidated evidence of the close relationship between plaques and activated glial cells, neuro-inflammation is increasingly believed to be an early player in the pathological cascade leading to AD rather than a mere consequence.

Although its role is still debated, the recognition of an inflammatory contribution to AD pathogenesis has led to therapeutic attempts using several anti-inflammatory agents [9]. Therapeutic approaches using phenols, phytoestrogens, neuro-steroids, and other natural phytochemicals have been explored in AD and experimental models, with some promising results such as cognitive improvements and attenuation of neuro-inflammation. Several non-steroidal anti-inflammatory drugs have also been tested in the attempt to prevent the onset or to slow down progression of AD [1, 2, 56, 57]. Flavonoids have been tested in AD models and seem to be able to reduce AD severity by modulating the production of microglia pro-inflammatory cytokines (TNF-α and IL-1β) or by reducing their Aβ-induced cytokine production [3]. In the present work, we tested IFNβ1a in an AD rat model and showed that its anti-inflammatory ability is mainly associated to the block of IL-1β and Il-6 upregulation levels in the hippocampus. Deposition of Aβ peptide may also activate astrocytes inducing astrogliosis with release of pro-inflammatory agents, such as IL-1, IL-6, and IL-10, and oxidative stress with production of reactive oxygen and nitrogen species [58, 59]. Changes in astrocyte function have been observed in brains from individuals with AD, as well as in AD in vitro and in vivo animal models [60]. However, in the present work, we could not observe a reduction of astrocytes activation in the Aβ_1-42_ group treated with IFNβ1a, as evaluated by the GFAP-positive cell number and GFAP protein levels.

IL-1β, synthesized and released by both activated microglia and astrocytes, is considered to be a major pro-inflammatory cytokine in the brain and play a key role in the progression of AD [61]. Similarly, IL-6 is also a pro-inflammatory cytokine mainly produced by activated microglia and when increased in brain of AD and AD animal models [5, 52] may impair cognitive processes, such as spatial learning and memory [62, 63], and stimulate the synthesis of Aβ precursor protein. Indeed, active microglia constitute the core immune system in the brain and release pro-inflammatory cytokines and free radicals that may elicit neurodegenerative processes, and since the pro-inflammatory mediators, IL-1β and Il-6, are produced primarily by microglia in the brain, the IFNβ1a inhibition of IL-1β and IL-6 in the hippocampus of Aβ_1-42_-treated group suggests a direct effect on microglial cells. This is supported by our observation of a reduction of microglial activation in the Aβ_1-42_ group treated with IFNβ1a, as reflected by the decrease in number of microglia cells and Iba-1 protein levels. Previously, following chronic exposure to IFNβ1a, Deczkowska et al. [48] reported modification in microglia morphology and expression of genes present in aged microglia, suggesting that microglia can contribute to the pro-inflammatory response of the brain in AD or aging, thereby exacerbating cognitive loss and disease pathology. In our rat model, we did not observe alterations in the microglia or astrocytes morphology, probably because the treatment time with IFNβ1a was shorter compared to those given by Deczkowska et al. [48]. In addition, in the present study, we did not explore gene expressed in aged microglia.

In contrast to hippocampal upregulation of IL-1 and IL-6 levels, the anti-inflammatory cytokines, IL-10 and TGF-β1, were significantly reduced in the Aβ_1-42_ group and recovered to control levels following IFNβ1a treatment. This result further supports the anti-inflammatory properties of IFNβ1a.

Although the numerous mechanisms underlying the IFNβ1a anti-inflammatory effects have been clearly defined within the peripheral immune system, its role in the central nervous system function has been little explored. Indeed, the only data available in this field, in addition to already mentioned anti-inflammatory effect of IFNβ1a in patients with multiple sclerosis [17, 18, 64], derived from studies on experimental autoimmune encephalomyelitis, an animal model of multiple sclerosis. Indeed, IFNβ1a treatment prevents and reduces the progression of the experimental CNS demyelination [42, 65, 66] by inhibiting pro-inflammatory cytokines (IL-6, IL-1β, TNF-α, IFN-γ), astrocytes activation, and inducible nitric oxide synthase expression [20]. However, adverse effect of type I interferon (IFN-I) has been reported that in the aging brain chronically elevated IFN-I activity contributes to the pathology of various human CNS diseases and in animal models, including aging microglial phenotype, neurodegeneration, and microgliosis [48].

The present and above-listed data showed beneficial effects of IFNβ1a in a model of AD pathology, but it is still unclear how it exerts this effect within the CNS, since its passage from the bloodstream to the brain parenchyma is significantly restricted by the blood–brain and blood–cerebrospinal fluid barriers [67–70]. In this context, the effects of IFNβ1a in the brain have been associated with possible modulation of brain inflammatory events at capillaries level [41] or with a decreased permeability of the blood–brain barrier to inflammatory cell entry into to the brain [71], thus reducing CNS inflammatory response. Several studies have demonstrated anti-inflammatory effect of IFNβ1a outside the CNS [17, 18, 64]. Indeed, IFNβ1a regulates several immunological functions, including decrease in T cell activation, induction of cytokine shifts in favor of an anti-inflammatory effect, prevention of T cell adhesion, and extravasation across the blood–brain barrier, as well as induction of T-regulatory cells, all occurring within the peripheral immunological organs [12–20]. Recently, several pathways for the transport of cytokines from systemic circulation into the brain have been reviewed [70], but the mechanisms by which IFNβ1a treatment may affect pro-inflammatory response induced by Aβ_1-42_ peptide injection in the hippocampus need further investigations. Anyway, in the present work, it is possible to exclude a role of glucocorticoid in mediating anti-inflammatory effects, since we did not observe any significant change in cortisol levels among the experimental groups.

Another particular hypothesis that has received considerable interest in the pathogenesis and progression of AD is the oxidative damage in the brain [72, 73]. Therefore, strategies aimed to reduce oxidative stress in AD have been proposed, also supported by reports of beneficial effects in AD of various antioxidants treatments [74, 75]. The present study indicated potential neuroprotective effects of IFNβ1a possibly mediated also by its ability to reduce ROS and lipids peroxidation and to increase SOD1 protein levels, although we did not find a parallel increase in SOD activity. A recent report actually showed that treatment with IFNβ1a inhibits oxidative stress in an animal model of multiple sclerosis, but at present, there are no reports of similar findings in AD or AD models [20].

## Conclusions

Based on the hypothesis that Aβ_1-42_-induced inflammation plays an important role in AD pathogenesis, using a rat model of AD, we provided evidence that IFNβ1a treatment may be a viable strategy to inhibit pro-inflammatory cytokines and oxidative stress. Therefore, IFNβ1a treatment, used for decades to contain inflammatory-mediated diseases of the brain, could be effective in AD patients, contributing to smolder the progression of this devastating disease.

## Abbreviations

AD: Alzheimer disease
DCF: Dichlorofluorescein
DCFH-DA: Dichlorofluorescein diacetate
ELISA: Enzyme-linked immunosorbent assay
IFNβ1a: Interferon-β1a
MDA: Malondialdehyde
NF-kB: Transcription factor nuclear factor-kB
PLSD: Fisher’s Protected Least Significant Difference
pNF-kB: Phosphorylated NF-kB
ROS: Reactive oxygen species
TBA: Thiobarbituric acid
TGF-β1: Transforming growth factor-beta1
Aβ_1-42_: Amyloid β Protein Fragment 1-42
